# Macrophage polarity and wound age determination

**DOI:** 10.1038/s41598-022-24577-9

**Published:** 2022-11-25

**Authors:** Yumi Kuninaka, Yuko Ishida, Akiko Ishigami, Mizuho Nosaka, Jumpei Matsuki, Haruki Yasuda, Ayumi Kofuna, Akihiko Kimura, Fukumi Furukawa, Toshikazu Kondo

**Affiliations:** Department of Forensic Medicine, Wakayama Medical University, 811-1 Kimiidera, Wakayama, 641-8509 Japan

**Keywords:** Medical research, Cell migration

## Abstract

We investigated the dynamics of the gene expression of M1 and M2 macrophage markers during skin wound healing in mice. Expression of M1-macrophage markers, such as *Il12a, Tnf, Il6, Il1b,* and *Nos2* was upregulated after wounding and peaked at 1 or 3 days after injury, and that of M2-macrophage markers such as *Mrc1, Cd163, Ccl17, Arg,* and *Tgfb1,* peaked at 6 days after injury. Consistent with these findings, using triple-color immunofluorescence analysis revealed that F4/80^+^CD80^+^ M1 macrophages were more abundant than F4/80^+^CD206^+^ M2 macrophages on day 3 in mouse wound specimens, and that M2 macrophages were prominently detected in day 6 wounds. For application in forensic practice, we examined macrophage polarization using human wound specimens. The average ratios of CD68^+^iNOS^+^ M1 macrophages to CD68^+^CD163^+^ M2 macrophages (M1/M2 ratios) were greater than 2.5 for the wounds aged 2–5 days. Out of 11 wounds aged 1–5 days, five samples had the M1/M2 ratios of > 3.0. These observations propose that the M1/M2 ratios of 3.0 would indicate a wound age of 1–5 days as the forensic opinion. This study showed that M1 and M2 macrophages in human skin wound might be a promising marker for wound age determination.

## Introduction

In forensic science, estimation of wound age is an important issue in routine autopsies because it provides essential information for crime scene reconstruction and determines the relationship between wound and cause of death^1–4^. Increasing evidence has shown that various biological substances can be useful markers for determining wound age^5–10^. The current study investigated changes in macrophage polarization during wound healing.

Wound healing is a complex process involving many different cells, extracellular matrix (ECM), cytokines, and specific interactions among them^7,11,12^. Inflammation and inflammatory cells play an important role in wound healing of the skin. The inflammatory response to tissue injury is characterized by a relatively rapid accumulation of large numbers of neutrophils, followed by infiltration of blood monocytes that have differentiated into tissue macrophages at the wound site. Neutrophils are confined to the early stages of wound healing, but macrophages persist throughout the wound healing process^13,14^.

Macrophages are heterogeneous and their phenotypes and functions are regulated by the surrounding microenvironment. Macrophages are usually present in two different subsets: M1 macrophages are pro-inflammatory, and M2 macrophages are anti-inflammatory and immunomodulatory. M1 macrophages are known as classical macrophages; they are induced by LPS and IFN-γ, and secrete inflammatory cytokines, such as TNF-α, IL-1, IL-6, and iNOS^15,16^. These secretions can kill infectious organisms such as bacteria, viruses, and malignant tumor cells, and the dead cells are phagocytosed by macrophages^17,18^. Thus, M1 macrophages are thought to be involved in the maintenance of human homeostasis through infection protection and anticancer effects. Regulation of M1 macrophage function is necessary because excessive immune response leads to chronic inflammation and inflammatory disease^19^.

M2 macrophages, on the other hand, are involved in tissue remodeling and immune tolerance^20^. M2 macrophages are induced by cytokines such as IL-4 and IL-13 through STAT6 activation^21^. They secrete IL-10, arginase (ARG), and TGF-β to suppress the inflammatory response^16^. In addition, they are also powerful phagocytic cells that act by removing debris and induce both wound healing and angiogenesis^16^. Thus, M2 macrophages play a role in the maintenance of organs and soft tissues and the regulation of immune balance. However, M2 macrophages also have drawbacks, as tumor-associated macrophages (TAMs) often have an M2 phenotype and are known to promote tumor progression^17^.

Both M1 and M2 macrophages are important for wound healing^22,23^. During the spontaneous healing process, neutrophils enter the tissue, followed by monocyte precursors, which migrate to macrophages after 48 h and become the primary cells of the wound tissue site^24^. The four-step process of wound healing (homeostasis, inflammation, proliferation, and remodeling) has a time lag of about 3–5 days, and the remodeling phase requires an intermediate stage of activation of resident fibroblasts and myofibroblasts for collagen production. Klar et al. demonstrated that during wound healing, the macrophage phenotype changes from a more proinflammatory (M1) in the early stages after injury to a less proinflammatory (M2) in the late stages in vivo^22^. Wound macrophages are still poorly understood but are known to play different roles at different stages of the healing process and can change their phenotype over time in response to microenvironmental signals^25–27^.

The purpose of this study was to investigate macrophage infiltration and their phenotype during the healing process of skin wounds in mice and humans, and to examine the utility of M1 and M2 macrophages as markers for wound age determination.

## Results

### Kinetics of M1- and M2-macrophage marker gene expression in mouse skin wounds

**Figure 1:**
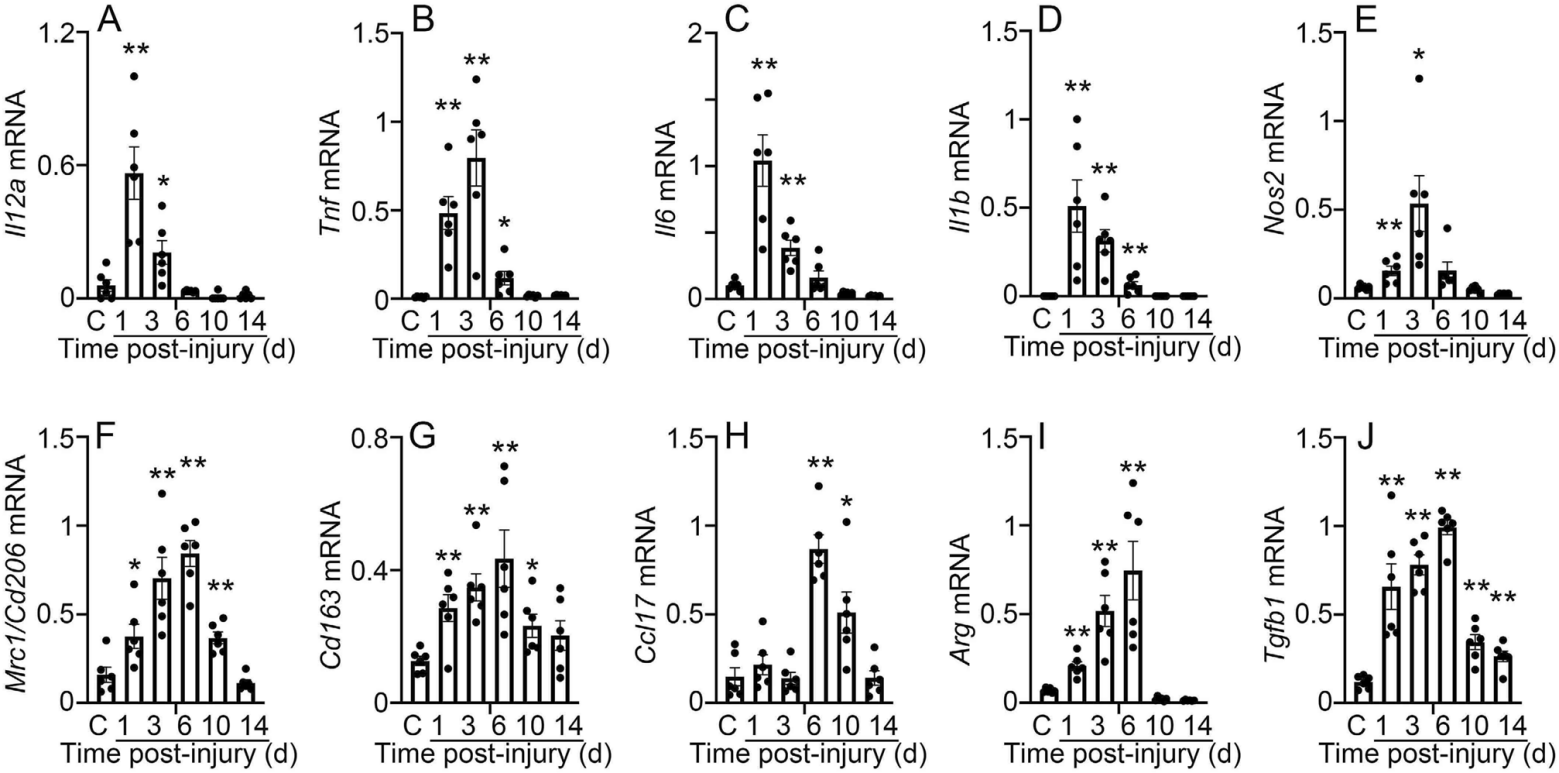
Expression kinetics of M1 and M2 macrophage markers in mouse skin wounds. M1: *Il12a* (**A**), *Tnf* (**B**), *Il6* (**C**), *Il1b* (**D**), and *Nos2* (**E**); M2: *Mrc* (**F**), *Cd163* (**G**), *Ccl17* (**H**), *Arg* (**I**), and *Tgfb1* (**J**). All values represent mean ± SEM. *P < 0.05, **P < 0.01, vs. controls.

Considering the important role of macrophage polarization in wound healing^22,23^, we determined the expression of M1- and M2-related genes in mouse skin wounds. Expression of M1-macrophage markers such as *Il12a, Tnf, Il6, Il1b,* and *Nos2* was upregulated after wounding and peaked at 1 or 3 days after injury (Fig. 1A–E). Expression of M1-macrophage markers was significantly enhanced in the early phase of wound healing, on the other hand, expression of M2-macrophage markers such as *Mrc1, Cd163, Ccl17, Arg,* and *Tgfb1* were peaked at 6 days after injury (Fig. 1F–J). These findings indicate that M1 macrophage infiltration during the early inflammatory phase and M2 macrophage infiltration during the proliferative phase are the prominent features of wound healing.

### Detection of M1- and M2-macrophages in mouse and human wound specimens

**Figure 2:**
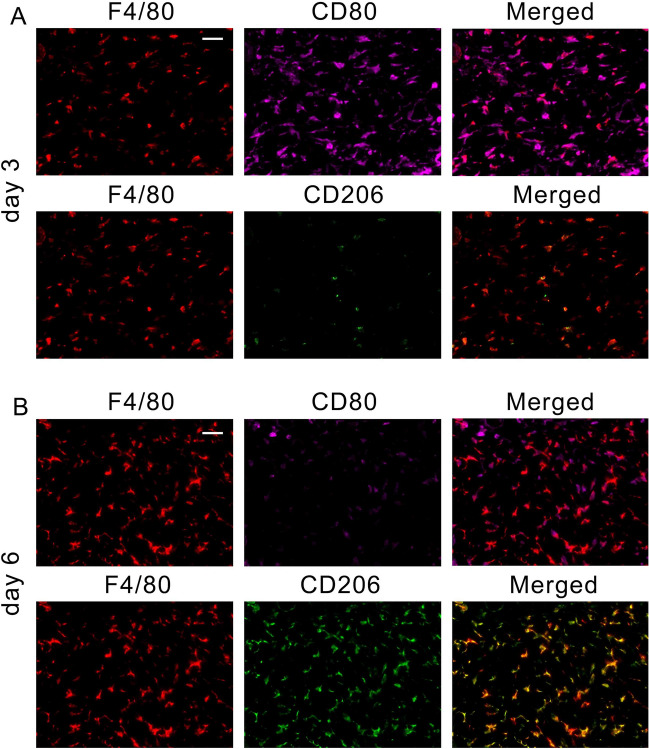
Emergence of F4/80^+^CD80^+^ M1 and F4/80^+^CD206^+^ M2 macrophages in mouse skin wounds. Triple-color immunofluorescence analysis was performed using anti-F4/80 (Cy3), anti-CD80 (Opal), and anti-CD206 (FITC). (**A**) Day-3 wound; (**B**) day-6 wound. Scale bar, 20 μm (×400).

**Figure 3:**
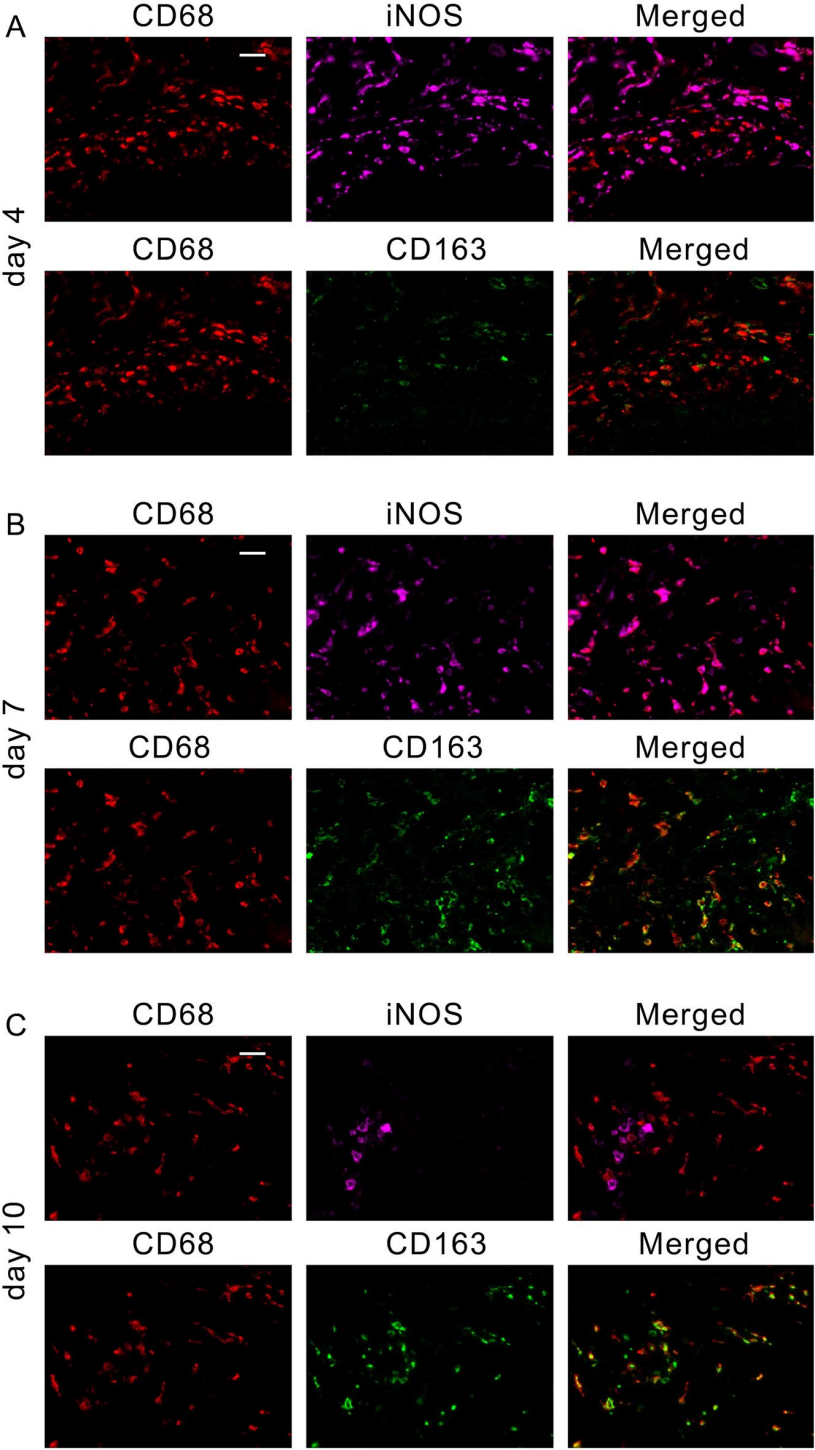

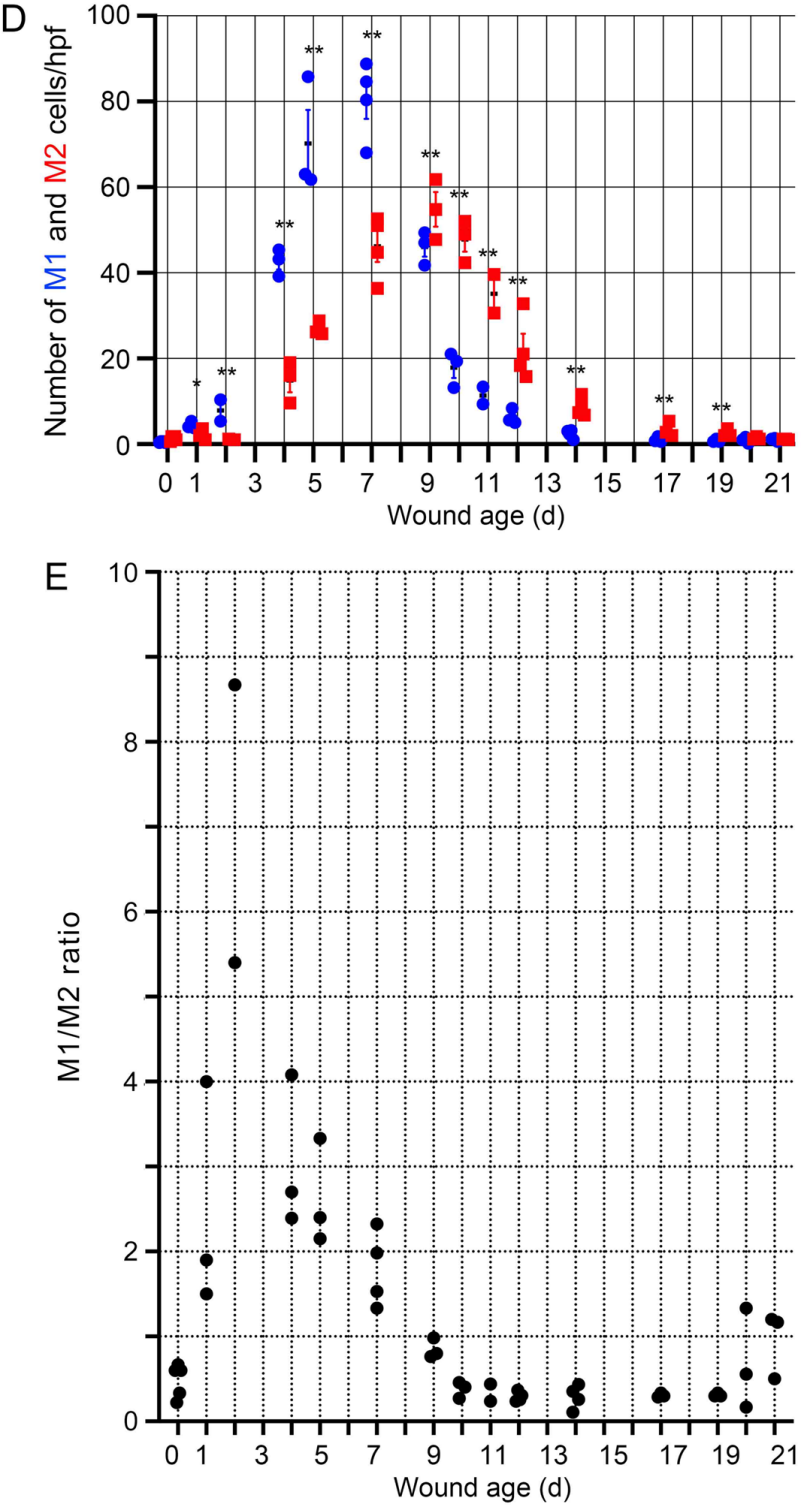
Emergence of CD68^+^iNOS^+^ M1 and CD68^+^CD163^+^ M2 macrophages in human skin wounds. (**A–C**) Triple-color immunofluorescence analysis was performed using anti-CD68 (Cy3), anti-iNOS (Opal), and anti-CD163 (FITC). (**A**) Day-4 wound; (**B**) day-7 wound; (**C**) day-10 wound. Scale bar, 20 μm (×400). (**D**) The number of M1 and M2 macrophages in human skin wounds. All values represent mean ± SEM. *P < 0.05; **P < 0.01, M1 vs. M2 macrophages. (**E**) Distribution of M1/M2 ratios associated with wound age.

Consistent with gene expression kinetics (Fig. 1), F4/80^+^CD80^+^ M1 macrophages were more abundant than F4/80^+^CD206^+^ M2 macrophages in 3-day mouse wounds (Fig. 2). In contrast, M2 macrophages were prominently detected in 6-day wounds in mice. For application in forensic practice, we examined macrophage polarization using human wound specimens. As expected, a large number of CD68^+^iNOS^+^ M1 macrophages was detected in the wound on day 4 of the early phase of repair, whereas the number of CD68^+^CD163^+^ M2 macrophages was low (Fig. 3A). Both human M1 and M2 macrophages were abundantly detected in the wounds on day 7 (Fig. 3B). M2 macrophages were predominantly present in wounds on day 10 of the proliferative phase of repair (Fig. 3C). In the morphometrical analysis, the number of M1 macrophages began to increase immediately after injury, peaked on day 7, and gradually decreased over time after injury (Fig. 3D). In contrast, M2 macrophages began to increase prominently on day 4 after injury, peaked on day 9, and then gradually decreased over time.

### M1/M2 ratios in human skin wounds

**Figure 4:**
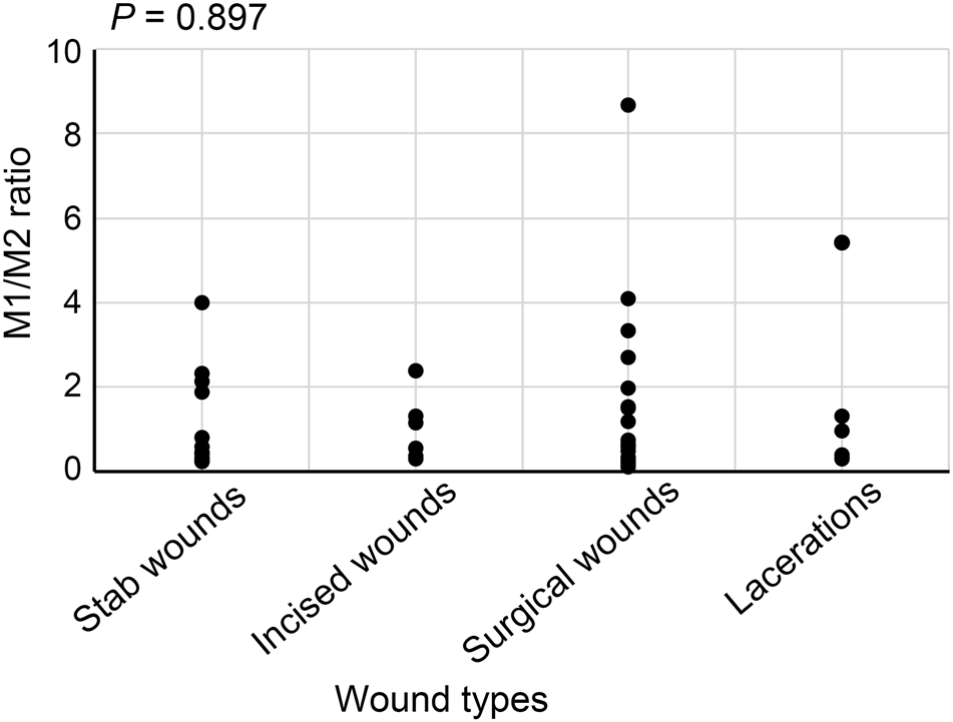
The relation between wound type and M1/M2 ratio in all cases. These results were obtained with Spearman’s correlation coefficient by rank test.

Comparing the numbers of M1 and M2 macrophages, the former was predominantly higher from 1 to 7 days after injury (Fig. 3E), with an M1/M2 ratio average of 1.79 ± 0.22 to 2.47 ± 0.78 (Fig. 3E and Table 1). Furthermore, all wounds aged 2–5 days showed M1/M2 ratios of > 2.0. In contrast, the number of M2 macrophages was higher than that of M1 macrophages and the average of M1/M2 ratio was less than 1.0 at nine or more days after skin injury and in few hours-wounds (Table 1). There was no significant difference in the M1/M2 ratio between the different types of wounds (Fig. 4).

**Table 1:** Means M1/M2 ratios in each wound age.

Age of wounds (days)	Mean ± SEM (range) of M1/M2 ratios
0	0.48 ± 0.09 (0.22–0.67)
1	2.47 ± 0.78 (1.50–4.00)
2	7.03 ± 1.63 (5.40–8.67)
4	3.06 ± 0.52 (2.39–4.08)
5	2.63 ± 0.36 (2.15–3.33)
7	1.79 ± 0.22 (1.33–2.32)
9	0.85 ± 0.07 (0.76–0.98)
10	0.38 ± 0.06 (0.27–0.46)
11	0.34 ± 0.10 (0.24–0.44)
12	0.29 ± 0.03 (0.24–0.37)
14	0.29 ± 0.07 (0.11–0.43)
17	0.31 ± 0.01 (0.29–0.33)
19	0.31 ± 0.01 (0.30–0.33)
20	0.69 ± 0.34 (0.17–1.33)
21	0.96 ± 0.23 (0.50–1.20)

## Discussion

To date, various techniques have been applied to forensic specimens to estimate wound age^28^. Given the assessment of wound vitality and age, the wound healing process is closely related to forensic pathology and dermatopathology. Evaluate innate immune cell, mesenchymal progenitor cell, and fibroblast kinetics to allow forensic estimation in the post-onset interval^29–33^. In addition, Kuninaka et al. have reported that dendritic cells may be a useful marker for determining wound age^34^. In the present study, we showed a biphasic phenotype of macrophages recruited to human and mice skin wounds.

Wound healing is a complex process involving the ECM, various cells, cytokines, and their interactions^35^. Macrophages are one of the central cells for successful wound healing^36,37^. They are known to secrete important cytokines and growth factors not only during the inflammatory phase but also during the proliferative phase of wound healing^38,39^. Wound macrophages in wounds are a critical source of important cytokines and chemokines during wound healing^40,41^. IL-6 plays a crucial regulatory role and is essential for efficient repair of skin injury^42^. The dynamics of IL-6, IL-1, and TNF-α levels are associated with the age of skin wounds^43^. Consistent with previous studies^44–47^, chemokines, such as IL-8, MCP-1, and MIP-1α, peaked within a few days after skin injury^48^. In addition, strong and sustained VEGF expression can be detected in human skin wounds and induces angiogenesis^49^.

Over the last decade, research has provided information on macrophage polarization. Macrophages can be been divided into M1 (classically activated) and M2 (alternatively activated) phenotypes^20,50–54^. Wound healing requires timely and sufficient M1 to M2 polarization, and inhibition of M1-M2 polarization has been shown to be closely associated with delayed wound healing^55^. In diabetic wound healing, there is excess of M1 and inadequate M2. In contrast, hypertrophic scars and keloids are overloaded with M2^56^. Therefore, the M1 and M2 phenotypes have different effects on wound healing. Moreover, Rodero et al. analyzed the subpopulation of wound-associated macrophages, and identified Ly6c^lo^MHCII^hi^ 'non-inflammatory' macrophages like M2 macrophages. This type of macrophages increased in both absolute number and proportion during normal wound healing and was absent in Ob/Ob and MYD88^-/-^ models of delayed healing^57^. Conversely, in a model of delayed wound healing in obese mice, the overexpression of inflammatory Ly6c^hi^MHCII^lo^ cells, like M1 macrophages, was observed^57^. We found differences in the appearance of M1 and M2 macrophages during the process of skin wound healing in mice using molecular biology and histopathological techniques.

During early wound healing, macrophages are actively mobilized to the wound site by cytokines produced by both surrounding cells and degradation products from pathogens^38^. At this stage, macrophages are considered inflammatory phenotype M1 and act primarily as phagocytes, releasing more cytokines and mobilizing more inflammatory cells^38^. In later stages, macrophages become polarized to the anti-inflammatory phenotype M2 and release cytokines essential for angiogenesis, cell migration, and ECM remodeling^38,39^. Timely resolution of each stage of wound healing and subsequent migration is important for successful wound healing^37^. Conversions of M1 and M2 macrophages and vice versa can be observed during infection, wound healing, and response to cancer^58,59^. LPS-preconditioned mesenchymal stem cell (MSC)-derived exosomes (LPS pre-Exo) promote diabetic skin wound healing by activating M2 macrophages^60^. Ti et al. demonstrated that LPS pre-Exo let-7b contributes to regulation of macrophage plasticity regulation, reduces chronic inflammation and improves skin wound healing^61^. The balance between these macrophage subpopulations is crucial for maintaining the physiological healing process^38,54,62^. Increased M1 macrophages, decreased M2 macrophages, and increased M2 activation can change the delicate balance of wound inflammation and have a dramatic impact on the repair process^63^. In this study, we found that both M1 and M2 macrophages showed continuous quantitative changes during wound healing in the human skin. M1 macrophages peaked on day 7 post-injury and M2 macrophages peaked on day 9 post-injury, consistent with the function of various macrophage subtypes: M1 macrophages play a pro-inflammatory role in early inflammation, while M2 macrophages promote tissue repair in late inflammation.

Several studies have shown that M2 macrophages may promote tissue fibrosis^64,65^. In fact, we also demonstrated that macrophages expressing CX3CR1 were also positive for the M2-specific marker CD206 in the lungs of the bleomycin-induced pulmonary fibrosis model^66^. These M2 macrophages were reduced in lungs of bleomycin-challenged, *Cx3cr1*^*-/-*^ mice. Moreover, in a skin carcinogenesis model, CX3CR1^+^ tumor-associated macrophages (TAMs) showed an M2 phenotype and abundantly expressed VEGF, a potent angiogenic factor^67^. CX3CR1 deficiency reduces M2 macrophage infiltration, VEGF expression, and ultimately suppresses skin carcinogenesis. Chen et al. have reported that M2 macrophages predominated in the wound tissues in the later stage and hypertrophic scar in the proliferative phase and were scattered throughout the dermis with high expression of the fibrous factor TGF-β1^68^.

M2 macrophages, also known as pro-fibrotic macrophages, secrete TGF-β and CTGF^69,70^. Fibrosis, in which M2 macrophages play a central role in the process of tissue repair, is common. Previous studies have shown that M2 macrophages are strongly associated with renal fibrosis^71^. Cardiac macrophages isolated from ischemic myocardial fibers are characterized by high expression of the M2 marker CD206^72^. Mesenchymal stem cells induce polarity to M2 and skin repair in non-healing wounds^73^. Furthermore, M2 macrophages promote fibrotic activity of human skin fibroblasts in vitro^74^. Wounds in diabetic patients showed dysregulated and persistent M1 macrophage polarization, whereas normal wounds show a transition to M2 macrophages around the third day after wounding^75^. The dominant macrophage population in day 5 mouse wounds has been reported to be the M2 macrophage population, demonstrating the effect of M2 on tissue remodeling^76^. The current study also demonstrated that M2-macrophage markers were upregulated and peaked 6 days after wounding (proliferative and remodeling stages of repair) in mice.

Macrophages have been reported to switch phenotype from M1 to M2 under certain conditions. Dermal fibroblasts could shift differentiation from inflammatory toward alternative macrophages^63^. M2 macrophages have been reported to be involved in angiogenesis and collagen synthesis related to the expression of VEGF, bFGF, and TGF-β^77,78^. Furthermore, M2 macrophage-derived exosomes can promote skin wound healing in situ by inducing direct conversion of M1 macrophages to an M2-like phenotype^79^. On the other hand, the typical shift from M1 macrophages to M2 macrophages observed in acute wounds was found to be uncontrolled in chronic wounds^63^. Thus, the promotion of polarization to M2 macrophages has been recognized as a novel mechanism to promote skin wound healing^80^.

Previous studies have shown that the *Nos2* to *Arg* mRNA ratio can be used as an indicator of M1/M2 activity balance^81,82^. The ratios of CD68^+^iNOS^+^ cells to CD68^+^CD163^+^ cells were measured to determine the dynamics of M1/M2 in human skin wounds. We found that the ratios of CD68^+^iNOS^+^ cells to CD68^+^CD163^+^ cells were > 1.0 in all wound samples with postinjury intervals between 1 to 7 days, whereas more than 9-day-old and few-hour-old wounds had the ratios of < 1.0. These observations suggest that the M1/M2 ratio of > 1.0 would indicate a wound age of 1–7 days. Out of 11 wounds aged 1–5 days, five samples had the M1/M2 ratios of > 3.0. These observations propose that the M1/M2 ratios of 3.0 would indicate a wound age of 1–5 days as the forensic opinion. Moreover, the M1/M2 ratios of > 4.0 would show a wound age of 1–4 days. In summary, differences in the number of M1 and M2 macrophages in human skin wounds may provide useful information for the determination of wound age. Although examining a single marker alone does not provide a high degree of confidence and objectivity in determining wound age, evidence suggests that immunohistochemical detection of M1 and M2 macrophages in skin wounds can provide important information for skin wound age determination. Combining several markers is likely to provide sufficient sensitivity and specificity.

## Methods

### Antibodies

The following monoclonal antibodies (mAbs) and polyclonal antibodies (pAbs) were used in this study: rat anti-mouse F4/80 mAb (1:50, clone BM8, T-2028, BMA Biomedicals, Switzerland); rat anti-mouse B7-1/CD80 mAbs (1:100, R&D Systems, clone, 111114; Minneapolis, MN); rabbit anti-mouse CD206 pAbs (1:1000, ab64693, Abcam, Cambridge, UK); mouse anti-human CD68 mAb (1:100, clone PG-M1, M0876, DAKO); rabbit anti-human iNOS pAbs (1:500, GTX124210, GeneTex, Alton Pkwy Irvine, CA); mouse anti-human CD163 mAb (1:200, clone, 10D6; Leica Biosystems, Buffalo Grove, IL); goat anti-mouse IgG (HRP) preabsorbed (1:200, ab97040, abcam, Cambridge, UK); goat anti-rabbit IgG (HRP) preabsorbed (1:200, ab7090, abcam); and goat anti-rat IgG (HRP) preabsorbed (1:200, ab7097, abcam).

### Animals

Male C57BL/6 mice were obtained from SLC Japan (Shizuoka, Japan). All male animals were used at 8–10 weeks of age and housed individually in cages under specific pathogen-free conditions during the experiment. All animals were used under the auspices of a protocol approved by the Wakayama Medical University.

### Excisional wound preparation and analysis

Full-thickness wounds were created on the back skin under sterile conditions^83^. Briefly, mice were anesthetized with i.p. administration of ketamine and xylazine. After shaving and cleaning with betadine and 70% ethanol, the dorsal skin was picked up at the midline and punched through two layers of skin with a sterile disposable biopsy punch (4-mm in diameter; Kai Industries, Tokyo, Japan), generating one wound on each side of the midline. The same procedure was repeated three times, generating six wounds. In some experiments, wounds and their surrounding areas, including the scab and epithelial margins, were cut at the indicated time intervals with a sterile disposable 8-mm diameter biopsy punch (Kai Industries), and were frozen with liquid nitrogen and stored at -80 ºC until RNA extraction and quantitative RT-PCR analysis as described below.

### Human skin wound samples

As shown in Table 2, forty-eight human skin wounds with various wound ages (wounds of few hours to 21 days were obtained from forensic autopsy cases with the postmortem interval of < 3 days^4,34^. This study included no cases of nutrition disorder, malignant/metabolic diseases, and glucocorticoid-taking. This study also included no wound considered to be contaminated and infected. Samples were fixed with 10% neutral-buffered formalin, and were then embedded with paraffin in order to prepare sections for immunofluorescence analyses as described below.

**Table 2:** Wound types.

Type	Number
Stab wounds	14
Incised wounds	7
Surgical wounds	21
Lacerations	6

Ages: 12 to 89 years (mean age, 55.9 years).

### Triple-color immunofluorescence analysis

Wound specimens were fixed in 4% formaldehyde buffered with PBS and then embedded with paraffin. Wound sections were analyzed by triple-color immunofluorescence microscopy as described previously^4,34^. Briefly, deparaffinized sections were incubated with PBS containing 1% normal donkey serum and 1% BSA to reduce nonspecific reactions. Then, the sections were further incubated with the combination of anti-mF4/80, -mCD80, and -mCD206 or anti-hCD68, -hiNOS, and -hCD163 Abs at 4 °C overnight. All Abs were used at a concentration of 1 μg/ml. After incubation with HRP-conjugated secondary Abs at room temperature for 30 min and TSA Plus kits (NELL741001KT, TSA Plus Fluorescein, AKOYA Biosciences, Marlborough, MA; or NEL8111001KT, Opal 7-color Manual IHC Kit, PerkinElmer, Waltham, MA) according to the manufacturer’s instruction, the sections were observed by fluorescence microscopy. As negative control, the sections were incubated with non-immunized rabbit IgG or mouse IgG.

### Real-time RT-PCR analysis

Total RNA was extracted from whole tissue using ISOGEN (Nippon Gene, Toyama, Japan), according to the manufacturer’s instructions. Total RNA was reverse transcribed to cDNA using PrimeScript Reverse Transcriptase (Takara Bio, Shiga, Japan) with oligo(dT)_15_ primers. First strand cDNA was synthesized from 2 μg total RNA with oligo(dT)_15_ primers using PrimeScript Reverse Transcriptase (Takara Bio). As described previously^84^, real-time PCR was carried out using SYBER Premix Ex Taq II (Takara Bio) with specific primer sets (Table 3). Primers were purchased from Takara Bio. Amplification and detection of mRNA were performed using Thermal Cycler Dice Real Time System (TP800; Takara Bio) according to the manufacturer’s instructions. To standardize mRNA concentrations, transcript levels of *Actb* were determined in parallel for each sample, and relative transcript levels were corrected by normalization based on *Actb* transcript levels.

**Table 3:** Sequences of primers used for real-time RT-PCR.

Transcript	Sequence
*Il12a*	(F) 5′-TGTCTTAGCCAGTCCCGAAACC-3′ (R) 5′-TCTTCATGATCGATGTCTTCAGCAG-3′
*Tnf*	(F) 5′-AAGCCTGTAGCCCACGTCGTA-3′ (R) 5′-GGCACCACTAGTTGGTTGTCTTTG-3′
*Il6*	(F) 5′-CCACTTCACAAGTCGGAGGCTTA-3′ (R) 5′-GCAAGTGCATCATCGTTGTTCATAC-3′
*Il1b*	(F) 5′-TCCAGGATGAGGACATGAGCAC-3′ (R) 5′-GAACGTCACACACCAGCAGGTTA-3′
*Nos2*	(F) 5′-GCAGAGATTGGAGGCCTTGTG-3′ (R) 5′-GGGTTGTTGCTGAACTTCCAGTC-3′
*Mrc*	(F) 5′-AGCTTCATCTTCGGGCCTTTG-3′ (R) 5′-GGTGACCACTCCTGCTGCTTTAG-3′
*Cd163*	(F) 5′-ACTTCAGAATCACATCATGGCACA-3′ (R) 5′-TCGTCGCTTCAGAGTCCACAG-3′
*Ccl17*	(F) 5′-TCAGTGGAGTGTTCCAGGGATG-3′ (R) 5′-GGCGTCTCCAAATGCCTCA-3′
*Arg*	(F) 5′-AGCTCTGGGAATCTGCATGG-3′ (R) 5′-ATGTACACGATGTCTTTGGCAGATA-3′
*Tgfb*	(F) 5′-TTCCGCTGCTACTGCAAGTCA-3′ (R) 5′-GGGTAGCGATCGAGTGTCCA-3′
*Actb*	(F) 5′-CATCCGTAAAGACCTCTATGCCAAC-3′ (R) 5′-ATGGAGCCACCGATCCACA-3′

(F), forward primer; (R), reverse primer.

### Morphometry

As reported previously, morphometry was carried out for a semiquantitative evaluation^34,85^. Briefly, five high-power microscopic fields (400×, 0.2 mm × 0.3 mm) were randomly chosen in each section, and the number of CD68^+^iNOS^+^ M1 macrophages and CD68^+^CD163^+^ M2 macrophages were counted, and the average number was evaluated in each wound specimen. The ratios of M1 macrophages to M2 macrophages (M1/M2 ratio) were calculated.

### Statistical analyses

The means of M1 and M2 macrophage numbers and the standard error (SE) were calculated. One-factor analysis of variance test was used followed by Scheffe’s *F* test. *P* < 0.05 was considered as significant.

### Study approval

All animal experiments were approved by the Committee on Animal Care and Use at Wakayama Medical University (No. 1082) and all methods were performed in accordance with relevant regulations and guidelines including the ARRIVE guideline. The human studies were approved and conducted in accordance with policies of the Research Ethics Committee of Wakayama Medical University (No. 3229). All procedures were carried out in accordance with the Declaration of Helsinki Principles. This study was conducted using autopsy records from the past, and we could not obtain informed consent from the bereaved family for the use of the records. Therefore, in accordance with the "Ethical Guidelines for Medical Research Involving Human Subjects (enacted by the Ministry of Health, Labor and Welfare in Japan)," Section 12-1 (2) (a) (c). The review board of Research Ethics Committee of Wakayama Medical University waived the need for written informed consent from relatives of individuals studied since this was a de-identified retrospective study of archived autopsy-derived tissue.

### Ethical approval

All procedures performed in studies involving human participants were in accordance with the ethical standards of the Japanese Society for Forensic Pathology, the Committee on Animal Care and Use at Wakayama Medical University, and the Research Ethics Committee of Wakayama Medical University.

## Data Availability

The authors declare that all data are available in the article file or available from the corresponding authors, Yuko Ishida and Toshikazu Kondo, upon reasonable request.

## Abbreviations

mAb: Monoclonal antibody
pAbs: Polyclonal antibodies
ECM: Extracellular matrix
ARG: Arginase
TAM: Tumor-associated macrophage
MSC: Mesenchymal stem cell
